# Genomic and Phenotypic Characterization of Two High-Risk *Klebsiella pneumoniae* Clones (ST258-blaKPC-2 and ST11-blaNDM-1) from a Greek Tertiary Hospital

**DOI:** 10.3390/antibiotics14111146

**Published:** 2025-11-12

**Authors:** Ilias S. Frydas, Emmanouil Kouklakis, Georgios Meletis, Andigoni Malousi, Maria Anna Kyriazidi, Fani Chatzopoulou, Irini Amargianitaki, Kallirhoe Kalinderi, Maria Mavridou, Stella Mitka, Evangelia Panagiotaki, Maria Chatzidimitriou

**Affiliations:** 1 Department of Biomedical Sciences, International Hellenic University, 57400 Thessaloniki, Greece; ifrydas@ihu.gr (I.S.F.); kallkali@ihu.gr (K.K.); mavridoumaria@ihu.gr (M.M.); mitka@ihu.gr (S.M.); 2 HERACLES Research Center on the Exposome and Health, Centre for Interdisciplinary Research and Innovation, Aristotle University of Thessaloniki, 57001 Thessaloniki, Greece; 3 Microbiology Department, General Hospital “Venizeleio-Pananio”, 71409 Irakleio, Greece; e.kouklakis@gmail.com (E.K.); eirini_amar@hotmail.gr (I.A.); evapanagiotaki7@gmail.com (E.P.); 4 Laboratory of Microbiology, School of Medicine, Aristotle University of Thessaloniki, 54124 Thessaloniki, Greeceandigoni@auth.gr (A.M.); 5 1st Propaedeutic Department of Internal Medicine, Medical School, Faculty of Health Sciences, Aristotle University of Thessaloniki, 54642 Thessaloniki, Greece; mkyriaza@auth.gr

**Keywords:** *Klebsiella pneumoniae*, carbapenemase, New Delhi metallo-β-lactamase-1, ST258, ST11, high-risk clones

## Abstract

**Background/Objectives**: *Klebsiella pneumoniae* ST258 and ST11 are global high-risk antimicrobial-resistant clones known for their virulence and resistance gene dissemination. This study aims to identify these clones in a Greek tertiary hospital and understand their resistance profiles and transmission dynamics. **Methods**: In January 2025, we isolated two distinct carbapenem-resistant *K. pneumoniae* in a Greek tertiary hospital: INT18S from an ICU patient’s bronchioalveolar lavage and INT20U from a urine sample in the emergency unit. Antimicrobial susceptibility testing (via Microscan system) and Whole-Genome Sequencing (WGS) were conducted on both isolates and their genomes were submitted to the NCBI. **Results**: The INT18S isolate carried the *bla*_KPC-2_ gene and belonged to the ST258 clone. The INT20U isolate carried the *bla*_NDM-1_ gene and belonged to the ST11 clone lineage. Both isolates contained at least one of the extended spectra β-lactamase genes tested (*TEM*, *SHV*, *OXA-1* and *CTX-M* group). **Conclusions**: The co-existence of the high-risk *K. pneumoniae* clones ST258 and ST11 in different hospital departments increases the risk of resistance gene transfer and suggests potential intra-hospital transmission pathways. Understanding their resistance profiles is critical for guiding treatment strategies and preventing the spread of multidrug-resistant pathogens.

## 1. Introduction

Klebsiella pneumoniae is a high-risk bacterial pathogen, which can lead to severe pneumonia and bloodstream and urinary infections [1,2]. Carbapenem-resistant K. pneumoniae (CRKP) strains—especially those producing carbapenemases, like KPC and NDM—pose a significant threat to public health due to their multidrug-resistant nature [3,4]. These carbapenemases, since they are mainly located on a variety of plasmids, facilitate horizontal gene transfer, contributing to the spread of resistance [5]. Infections caused by CPKP are associated with high mortality rates, but certain enzymes like NDM and VIM that confer resistance to a broader range of drug classes may be linked to even worse patient outcomes [3,4,5]. These enzymes often coexist with extended-spectrum β-lactamases (ESBLs) or other resistance factors, leading to extensively drug-resistant (XDR) or even pan-drug-resistant (PDR) phenotypes [6].

Strain-specific *K. pneumoniae* has been shown to exhibit within-host diversity during prolonged infections with certain variants persisting through adaptation [7]. Notably also, CRKP isolates from the blood of a single patient have shown heterogenicity in their antibiotic susceptibility, capsular polysaccharide production and mucoviscosity [8,9,10]. These CRKP strains frequently isolated in ICUs and other hospital settings are associated with prolonged hospitalization, increased mortality rates and limited treatment options [11].

The majority of CRKP strains worldwide belong to the notorious clonal complex 258 CC258, including sequence types (STs) ST258, ST11, ST340, ST437 and ST512 [12]. These strains combine invasive virulence features with resistance to last-resort antibiotics and have been linked to both community-and hospital-associated outbreaks [13].

In Greece, the epidemiological landscape of *K. pneumoniae* is dominated by CC258, particularly sequence types ST258 and ST11. Several studies have documented the isolation of *K. pneumoniae* ST258 strains with similar resistance profiles in Greece [14,15]. These strains are often associated with the *bla*_KPC-2_ gene and various plasmid types, contributing to their multidrug resistance. A study analyzed 378 *K. pneumoniae* isolates collected from 40 Greek hospitals between January 2009 and April 2010 and found that all isolates were positive for *bla*_KPC-2_, with ST258 being the predominant sequence type (*n* = 322) [15]. Another comprehensive survey covering the period from 2013 to 2022 identified ST258/512 as one of the five most prevalent sequence types among carbapenem-resistant *K. pneumoniae* isolates in Greek hospitals [16]. Despite this evidence, the genomic context and intra-hospital transmission dynamics of these high-risk clones remain underexplored.

In the present study we aimed to elucidate the molecular and transmission characteristics of two separate *K. pneumoniae* isolates belonging to the high-risk clones ST258 and ST11, each carrying distinct carbapenemase genes (*blaKPC-2* and *blaNDM-1*) co-existing in the same tertiary hospital in Irakleio, Crete, Greece. This co-occurrence suggests possible intra-hospital transmission pathways that have not been previously explored. Also, by focusing on these two high-risk clones from different hospital departments, we aim to deepen the understanding of their genetic backgrounds and transmission potential, guiding the infection control and antibiotic stewardship strategies.

## 2. Results

### 2.1. Isolate Identification

**Figure 1:**
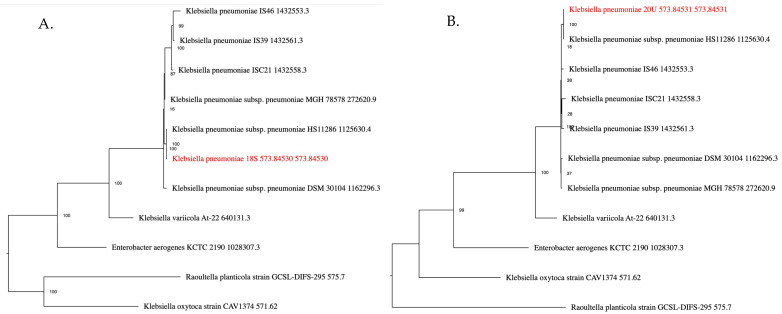
The National Center for Biotechnology Information (NCBI) staff manually select and categorize reference and representative genomes, which they consider to be of high quality and importance to the research community. PATRIC provides the reference and representative genomes, and includes them in the phylogenetic analysis that is part of the Comprehensive Genome Analysis report. The closest reference and representative genomes were identified by Mash/MinHash^.^ PATRIC global protein families (PGFams) were selected from these genomes to determine the phylogenetic placement of this genome. The protein sequences from these families were aligned with MUSCLE, and the nucleotides for each of those sequences were mapped to the protein alignment. The joint set of amino acid and nucleotide alignments were concatenated into a data matrix, and RaxM was used to analyze this matrix, with fast bootstrapping used to generate the support values in the tree. INT18S and INT20U isolates are displayed in red color in (**A**) and (**B**) respectively.

Whole-Genome Sequencing (WGS) identified the isolates as *K. pneumoniae* INT18S and INT20U, with a genome completeness of 97.5% and 97.85%, respectively, assigned at taxid 573. The assembly resulted in 147 contigs for INT18S and 137 contigs (≥500 bp) for INT20U, with N50values of 138,249 and 124,403 and total contig lengths of 5,684,851 and 5,551,402 bp, respectively, ensuring high-quality assemblies. The average sequencing depth was 185.4× for INT18S and 225.9× for INT20U. Multilocus sequence typing (MLST) showed that the INT18S isolate belongs to the ST258 clone, and the INT20U isolate belongs to the ST11 clone. Phylogenetic trees of both isolates are shown in Figure 1. Both isolates were predicted as capsule-null, with the K-locus 106 (KL106/O2v2) and K-locus 48(KL48/O2v1) detected for INT18S and INT20U, respectively.

### 2.2. Antimicrobial Resistance Genes (ARGs)

All the detected ARGs in both isolates are shown in Table 1. In the two isolates, different ARGs were detected, and the common ones were the *aac(6′)-lb*, conferring resistance to aminoglycosides, and the *OqxA* and *OqxB* which confer resistance to fluoroquinolones. INT18S carried a total of 13 ARGs, while INT20U carried 11 ARG, with 3 ARGs shared between them. This was determined using PATRIC’s k-mer-based ARGs detection method. The detection was further validated using AMRFinderPlus to ensure accuracy.

The *aadA2*, *aph(3′)-la*, *qacE*, *sul1*, *catA1*, *bla*_KPC-2_, *bla*_SHV-12_ and *mph(A)* genes were detected only in INT18S, and the *bla*_CTX-M-15,_ *bla*_NDM-1_, *bla*_OXA-1_, *bla*_SHV-182_, catB3 and dfrA14 genes were detected only in INT20U. The *bla*_KPC-2_ gene that was identified in INT18S encodes the predominant carbapenemase in Greece, particularly among ST258 strains, and is strongly associated with healthcare-associated outbreaks. The *bla*_SHV-12_ gene is an extended-spectrum β-lactamase (ESBL) that confers resistance to third-generation cephalosporins and is often co-transferred with carbapenemases. Aminoglycoside-modifying genes such as *aac(6′)-Ib*, *aac(6′)-Ibcr*, *aadA2* and *aph(3′)-Ia* provide high-level resistance to aminoglycosides, further limiting treatment choices. Other resistance determinants found, such as *catA1* (chloramphenicol), *dfrA12* (trimethoprim), *fosA6* (fosfomycin), *mph(A)* (macrolides), *qacE* (quaternary ammonium compounds) and *sul1* (sulfonamides), contribute to multidrug resistance.

### 2.3. Antimicrobial Susceptibility

The susceptibility of isolates to common antibiotics used in hospitals was tested, including third- and fourth- generation cephalosporins, β-lactam/β-lactamase inhibitors, cephamycin, aztreonam, quinolones, aminoglycosides and colistin. The antibiotic susceptibility profiles of *K. pneumoniae* INT18S and INT20U are detailed in Table 2. Both strains exhibited a resistance to carbapenems (ertapenem, imipenem and meropenem), amikacin, aztreonam, amoxicillin/clavulanate, ampicillin, chloramphenicol, cefuroxime, ceftazidime, cefoxitin, cefepime, ciprofloxacin, levofloxacin, piperacillin/tazobactam, trimethoprim/sulfamethoxazole, nalidixic acid and tobramycin. INT20U has been shown to be resistant to gentamicin, ceftazidime/avibactam, imipenem/relebactam and meropenem/vaborbactam whereas INT18S was susceptible. Both isolates remained susceptible to colistin and aztreonam/avibactam. European Committee on Antimicrobial Susceptibility Testing (EUCAST) breakpoints were applied for the interpretation of the susceptibility testing results of antibiotics (https://www.eucast.org/clinical_breakpoints, accessed on 20 September 2025).

### 2.4. Plasmid Detection and Additional Genomic Features

The in-silico predictions for the plasmid typing, including the identification of replicon, relaxase and mate-pair formation protein types for INT18S and INT20U, are shown in Table 3. The IncFIB(K) is the only common plasmid detected in both isolate genomes.

In INT18S, three ColRNAI plasmids were detected, which are notable for their ability to be mobilized and participate in plasmid fusion events, but no respective plasmids were detected in the INT20U isolate. In addition, three relaxase-type genes were detected in INT18S, denoted as MOBC, MOBF, and MOBP, and only MOBF was detected in INT20U. All plasmids were denoted as conjugative mobilizable types. The detection of ColRNAI, IncFIB(K), IncFIB(pQil) and IncX3 plasmids suggests an efficient horizontal transfer of resistance determinants through highly mobile genetic elements. Relaxase and conjugation systems and the presence of relaxase types MOBC, MOBF and MOBP, together with the MPF_T conjugation system, strongly indicate that the isolates are conjugative and capable of horizontal gene transfer.

### 2.5. Yersiniabactin

The sequencing data of the two strains were subjected to a Kleborate v3.1.3 analysis for Yersiniabactin sequence type (YbST) and Yersiniabactin Lineage ICE (ICEKp) predictions. Allele numbers of different yersiniabactin (ybt) locus and lineage results are shown in Table 4, with notable differences. It was found that the INT18S isolate carried the yersiniabactin gene cluster ybt13 and the respective encoding integrative conjugative element ICEKp2. INT20U carried the ybt gene cluster *ybt15* and the respective integrative conjugative element ICEKp11. In isolate INT18S, three to six times more alleles for *irp2*, *irp1* and *ybtE* genes were identified, whereas isolate INT20U showed four times more alleles for the *ybtX* gene and six times more alleles for the *ybtU* gene. These differences could imply enhanced iron acquisition and survival capabilities in the host, contributing to the pathogenicity of INT18S. A resistance score three for INT18S indicates a higher potential for virulence compared to a score of two for INT20U. These scores are based on the presence of carbapenemase genes and colistin resistance determinants, and provide a quantitative measure of the virulence potential, reflecting the genetic composition of the isolates’ yersiniabactin clusters [17].

## 3. Discussion

This study conducted a molecular and phenotypic analysis of two distinct clinical *K. pneumoniae* isolates obtained from different departments in a Greek hospital. The use of WGS techniques enabled the precise identification of ST258 and ST11, revealing complex resistance profiles and potential intra-hospital transmission dynamics.

Our findings are consistent with previous reports of high carbapenem resistance rates in Greece, driven largely by the spread of *bla_KPC-2_* in ST258 clones. Between 2001 and 2008, Greece reported some of the highest rates of carbapenem resistance in Gram-negative bacteria globally, with the carbapenemase prevalence increasing by 30% in hospital wards and 60% in intensive care units (ICUs) [18,19]. A study performed in 21 Greek hospitals indicated that by the end of 2008, 96% of *K. pneumoniae* isolates carrying *bla*_KPC_ belonged to the ST258 lineage which accounted for nearly 40% of all isolates [20]. The surveillance in Greece from 2013 to 2022 has demonstrated that *bla*_KPC_, originally carried almost exclusively by the hyperepidemic ST258 clone, now circulates across numerous STs, including ST147, ST323 and three other high-risk clones (ST11, ST15 and ST39), representing over 90% of the CRKP in Greek hospitals [16,21,22,23]. The surveillance by the European Centre for Disease Prevention and Control (ECDC) between 2022 and 2023 involving 15 Greek hospitals confirmed that the emerging high-risk clones ST39 and ST323 have become endemic, across the hospital network and in multiple health institutions, respectively [24]. Another study conducted in an ICU in Thessaloniki performed between 2016 and 2019 with 165 patients, demonstrated the polyclonal transmission of *bla_KPC_* identifying 128 pulsotypes within 17 clusters, and found that 74% of CRKP isolates harbored *bla*_KPC_ genes [25].

The present study provides a detailed molecular and phenotypic analysis of two novel isolated carbapenem-resistant *K. pneumoniae* isolates, collected at a regional hospital in Crete, Greece, and annotated as INT18S and INT20U. Our findings reveal a complex and evolving epidemiological landscape of CRKP in Greek hospitals, with a predominance of high-risk clones ST258 and ST11, extensive resistance gene co-carriage and potential intra-hospital transmission. *K. pneumoniae* strains in ICUs and emergency units can differ in terms of virulence, antibiotic resistance and the types of infections they cause [26]. Strains isolated from ICU settings are typically linked with hospital-acquired infections, including CRKP, and can lead to severe respiratory and bloodstream infections with high morbidity and mortality rates. On the other hand, emergency unit strains, while also capable of causing infections, may be more associated with community-acquired pneumonia and other infections and often display distinct antibiotic resistance profiles [27]. The identification of CRKP in the Emergency Department suggests possible healthcare-associated transmission from prior hospital stays or nursing home residents, whereas the co-existence of multiple carbapenemases reflects ongoing gene exchange and selective pressure.

In the current study, it was determined that the INT18S and INT20U *K. pneumoniae* isolates belong to the ST258 and ST11 sequence types, respectively. The latter types belong to clonal complex 258 (CC258), but they differ primarily in their genomic makeup and more specifically at the region containing the capsule polysaccharide (cps) gene [28]. ST258 is considered a hybrid clone, with a ST11 backbone and a segment from ST442, including the cps operon, whereas ST11 is a single-locus variant of ST258 [29]. While ST258 is prevalent in North America, Latin America and Europe, ST11 dominates in Asia, and especially in China. Furthermore, ST11 isolates have shown higher serum resistance and phagocytic rates compared to ST258 [30]. It was shown that the ICU-detected INT18S isolate is resistant to carbapenems, which is consistent with data supporting the strong resistance of ST258 clonal populations in tertiary hospitals due to integrative conjugative elements such as ICEKp10 and the harboring of yersiniabactin and colibactin virulence factors [31]. INT18S showed more yersiniabactin alleles than INT20U. The detection of yersiniabactin (ybt) loci in *K. pneumoniae* isolates is significant because it is a major virulence factor that facilitates iron acquisition, a critical process for bacterial growth and survival in the host. Moreover, it is often found in highly pathogenic and multidrug-resistant strains, and its presence can indicate a higher risk of severe invasive infections, such as bacteremia and liver abscesses [31,32].

The *K. pneumoniae* INT18S isolate exhibits a concerning resistance profile that is characteristic of epidemic clones responsible for nosocomial infections in Greece [33]. Its resistome includes *bla*_KPC-2_ together with multiple resistance genes such as *aac(6′)-Ib*, *aac(6′)-Ibcr*, *aadA2*, *aph(3′)-Ia*, *bla*_SHV-12_, *catA1*, *dfrA12*, *fosA6*, *mph(A)*, *OqxA*, *OqxB*, *qacE* and *sul1*, which confer resistance to aminoglycosides, fosfomycin, macrolide, sulfonamide and β-lactams. The latter are hosted on diverse plasmid types with known mobility functions, such as ColRNAI, IncFIB(K), IncFIB(pQil) and IncX3, indicating high transmissibility; dissemination dynamics within and between species, particularly in healthcare environments; and severely limited treatment options [34,35,36,37]. In parallel, the coexistence of multiple plasmid replicons with relaxase systems (MOBF, MOBC and MOBP) supports a high potential for horizontal gene transfer. Multireplicon plasmids, such as IncFIB variants, often carry multiple resistance operons, increasing the genetic payload and potentially enabling co-selection under antibiotic pressure (e.g., using aminoglycosides also selects for β-lactam resistance) [4]. This constellation of genes and plasmids likely contributes to persistence and spread in hospital environments, especially in high-antibiotic-pressure settings such as ICUs.

On the other hand, the urine-derived isolate INT20U of a patient admitted to the Emergency Department was assigned to ST11, another pandemic lineage often associated with *bla_NDM-1_* and *bla*_CTX-M-15_ dissemination, particularly in Asia, the Balkans and parts of the Mediterranean [38]. This isolate harbored *bla*_NDM-1_ together with class A β-lactamase *bla*_OXA-1_ and *bla*_SHV-182_. Interestingly, INT20U did not exhibit porin gene mutations, suggesting that its carbapenem resistance was primarily enzyme-mediated. Its plasmid content (IncFIA(HI1), IncFII(K) and, IncR) supports a distinct mobilome compared with INT18S and may indicate exposure to different ecological niches, reservoirs or selective pressures, including community or early healthcare settings [39]. Phenotypically, INT20U showed resistance to ceftazidime/avibactam and imipenem/relebactam, aligning with the typical drug resistance pattern of NDM-producing isolates and emphasizing limited therapeutic options.

Our study identified the presence of the IncFIB(K) plasmid replicon in both INT18S and INT20U *K. pneumoniae* isolates. The co-occurrence of this plasmid in both isolates, despite their origin from different departments (ICU and emergency unit) suggests a potential mechanism for the local transmission of resistant genes. IncFIB(K) is well known for its high mobility due to its conjugative properties. This capability is further augmented by the presence of MOBF relaxase, which was also detected in our isolates [14].

Neither isolate harbored hypervirulence-associated rmp loci (*rmpA* and *rmpD*) excluding a hypermucoid *K. pneumoniae* phenotype and indicating that their capsule-null phenotype may enhance high adaptability and tissue tropism affecting virulence and resistance dynamics [15,40]. This adaptability may facilitate strain evolution and increased transmission rates [41,42].

This study has some limitations. Additional experiments and analyses are required, such as conjugation experiments to assess the horizontal transfer potential of the resistance plasmids. Also, the isolates were obtained from two separate patients in a tertiary hospital setting, raising the possibility of selection bias and limiting the generalization of our findings.

In conclusion, CRKP in this regional hospital in Crete, Greece shows diverse resistance profiles. The presence of both ST258 harboring *bla*_KPC-2_ and ST11 harboring *bla*_NDM-1_ lineages highlights the need for enhanced molecular diagnostics, active genomic surveillance, and strict antimicrobial stewardship programs in Greek hospitals and beyond. These are critical to mitigating spread and improving patient outcomes.

## 4. Materials and Methods

### 4.1. Collection and Identification of Bacterial Strains

The *K. pneumoniae* strain INT8S was isolated from bronchial secretion of a male patient who was hospitalized in the intensive care unit (ICU) of a Greek tertiary Hospital in Irakleio, Crete, Greece. The patient with underlying hematologic disease and kidney failure was treated with ceftazidime and teicoplanin. Four days after sampling the patient passed away. The *K. pneumoniae* strain INT20U was isolated from urine sample of a male patient admitted to the Emergency Department of the hospital. At admission, the patient was treated with ampicillin/sulbactam (500 + 1000 mg), twice a day.

The hospital has granted ethical approval (5/6 May 2025), and patients’ samples were further processed in the hospital’s Microbiology Department and cultured in routine media. Antimicrobial susceptibility testing was carried out by the Microscan Walk away 96 plus automated system (Beckman Coulter, Brea, California, USA) according to manufacturer’s instructions. Susceptibility testing for newer antimicrobial combinations of ceftazidime/avibactam, aztreonam/avibactam, imipenem/relebactam and meropenem/vaborbactam was performed using minimal inhibitory concentration (MIC) gradient strips (Liofilchem Roseto degli Abruzzi, Italy). Moreover, susceptibility to colistin was tested using a cation adjusted Mueller–Hinton broth (CAMHB) microdilution method (Liofilchem, Roseto degli Abruzzi, Italy). Tigecycline susceptibility was evaluated using susceptibility breakpoints (susceptible ≤ 2mg/L, intermediate 4 mg/L, resistant ≥ 8 mg/L), approved by the US Food and Drug Administration. European Committee on Antimicrobial Susceptibility Testing (EUCAST) breakpoints were applied for the interpretation of the susceptibility testing results of all other antibiotics (https://www.eucast.org/clinical_breakpoints, accessed on 20 September 2025). Both isolates were phenotypically tested for metallo-β-lactamase (MBL) and *K. pneumoniae* carbapenemase (KPC) production using ethylene diamine tetraacetic acid (EDTA) and phenylboronic acid (BA).

### 4.2. DNA Isolation and Real-Time PCR Gene Detection

Genomic DNA was extracted from overnight bacterial culture using the PureLink Genomic DNA Mini Kit (Invitrogen, Carlsbad, CA, USA), according to the manufacturer’s protocol for Gram-negative bacteria. Detection of resistance genes (*KPC*, *VIM*, *NDM* and *OXA-48*) was conducted by real-time polymerase chain reaction (qPCR) using the *QuantStudio™ 5 Real-Time PCR System* (Applied Biosystems, Fostercity, CA, USA) in combination with the *Platinum^®^ Quantitative PCR SuperMix-UDG* (Invitrogen, Carlsbad, CA, USA). This master mix included uracil-N-glycosylase (UNG) to prevent carryover contamination from previously amplified PCR products containing uracil. Each reaction was prepared according to the manufacturer’s guidelines, with final concentrations of 40 nM for each primer and probe. Primers and probes were selected based on the study by Ellington et al., 2016 [43] and are shown in Table 5. The amplification protocol followed the same reference with minor modifications, consisting of an initial UNG activation step at 50 °C for 2 min, initial denaturation at 95 °C for 2 min, followed by 40 amplification cycles of denaturation at 95 °C for 15 s and annealing/extension at 60 °C for 1 min. A final extension step was performed at 60 °C for 5 min. Data analysis was carried out using the QuantStudio™ Design and Analysis Software. For quality control, a no-template control (NTC) and positive control DNA from well-characterized strains harboring each resistance gene were included in every run.

### 4.3. Whole-Genome Sequencing

Libraries were prepared using Ion Torrent technology and Ion Chef workflows (Thermo Scientific, Waltham, MA, USA). Sequencing was performed in the S5XLS system, and analysis of the raw sequencing data was conducted by Ion Torrent Suite v.5.10.0, according to manufacturer’s instructions. Raw sequencing data were quality checked and trimmed by fastp to keep only the reads that match the minimum length and quality criteria.

### 4.4. Bioinformatics Analysis

Libraries were prepared using Ion Torrent technology and Ion Chef workflows (Thermo Scientific). Sequencing was performed in the S5XLS system and analysis of primary data was conducted with Ion Torrent Suite (v.5.10.0). Kraken2 v2.13 was used for the taxonomy classification built on the standard database that includes NCBI taxonomic information, complete RefSeq microbe genomes, human genomes and a vector collection. Genomes were de novo assembled by Assembler SPAdes and SPAdes v3.15.5 using the default settings for the k-mers and the Ion Torrent configuration. BUSCO v5.7.1 (lineage: enterobacterales_odb10) was used to evaluate the assembled genome completeness of the synthesized genomes. PATRIC BV-BRC platform v3.54.6 was used to implement a comprehensive analysis of the genomes, including quality control, genome annotation, AMR detection and phylogenetic analysis. Complementary analysis for quality assessment and strain annotation was applied using tools such as QUAST v5.3.0 for assembly QC, staramr v0.10.0 for AMR validation, Kaptive v1.3.0 and kleborate v3.1.3. MOB-Suite v3.0.1 was used to determine plasmid replicon types and predict plasmid mobility. Finally, after they were trimmed of foreign species fragment contigs and converted to fasta files, bacterial genomes were submitted to NCBI genome database and received the respective accession numbers JBRBEL000000000 for INT18S and JBPXMH000000000 for INT20U, respectively.

## Figures and Tables

**Table 1:** Antimicrobial resistance (AMR) genes annotated in both INT18S and INT20U isolates using the k-mer-based AMR gene detection method of the Genome Annotation Service in PATRIC. Gene names in **boldface** denote genes detected in both isolates, and antibiotic names in **boldface** denote antibiotic resistance phenotypes predicted for both isolates.

Isolate	MLST	AMR Genes	Center for Genomic Epidemiology—DTU-Predicted Resistance Phenotypes
INT18S	ST258	***aac(6′)-Ib***, ***aac(6′)-Ibcr***, ***fosA6***, ***OqxA***, ***OqxB***, *aadA2*, *aph(3′)-Ia*, *bla*_KPC-2_, *bla*_SHV-12_, *catA1*, *dfrA12*, *qacE*, *sul1*, *mph(A)*	**Amikacin**, **Tobramycin**, **Netilmicin**, **Fluoroquinolones**, **Amoxicillin**, **Amoxicillin/Clavulanic Acid**, **Ampicillin**, **Ampicillin/Clavulanic Acid**, **Aztreonam**, **Cefepime**, **Cefotaxime**, **Cefoxitin**, **Ceftazidime**, **Ertapenem**, **Imipenem**, **Meropenem**, **Piperacillin**, **Piperacillin/Tazobactam**, **Ticarcillin**, **Ceftriaxone**, **Chloramphenicol**, **Trimethoprim**, **Fosfomycin**, **Nalidixic Acid**, Streptomycin, Neomycin, Kanamycin, Ticarcillin/Clavulanic Acid, Erythromycin, Azithromycin, Sulfamethoxazole
INT20U	ST11	***aac(6′)-Ib***, ***aac(6′)-Ibcr***, ***fosA6***, ***OqxA***, ***OqxB***, *aac(6′)-Ib-cr*, *bla*_CTXM-15_, *bla*_NDM-1_, *bla*_OXA-1_, *bla*_SHV-182_, *catB3*, *dfrA14*	**Amikacin**, **Tobramycin**, **Netilmicin**, **Fluoroquinolones**, **Amoxicillin**, **Ampicillin**, **Aztreonam**, **Cefepime**, **Cefotaxime**, **Ceftazidime**, **Ceftriaxone**, **Piperacillin**, **Ticarcillin**, **Amoxicillin/Clavulanic Acid**, **Ampicillin/Clavulanic Acid**, **Cefoxitin**, **Ertapenem**, **Imipenem**, **Meropenem**, **Piperacillin/Tazobactam**, **Chloramphenicol**, **Trimethoprim**, **Fosfomycin**, **Nalidixic Acid**, Cefixime, Temocillin

**Table 2:** Antibiotic susceptibility of *K. pneumoniae* INT18S and INT20U isolates. R: Resistant, S: Susceptible and MIC: Minimum Inhibitory Concentration. Differences in susceptibilities between the two isolates are highlighted in yellow.

	INT18S		INT20U	
Antibiotic	MIC (mg/L)	Interpretation	MIC (mg/L)	Interpretation
Amikacin	>16	R	>16	R
Amoxicillin/Clavulanate	>8/4	R	>8/4	R
Ampicillin	>8	R	>8	R
Aztreonam	>4	R	>4	R
Cefepime	>8	R	>8	R
Cefotaxime	>16	R	>16	R
Cefoxitin	>8	R	>8	R
Ceftazidime	>8	R	>8	R
Cefuroxime	>8	R	>8	R
Chloramphenicol	>8	R	>8	R
Ciprofloxacin	>1	R	>1	R
Colistin	≤2	S	≤2	S
Ertapenem	>1	R	>1	R
Gentamicin	≤2	S	>4	R
Imipenem	>8	R	>8	R
Levofloxacin	>1	R	>1	R
Meropenem	>8	R	>8	R
Nalidixic Acid	>16	R	>16	R
Nitrofurantoin	>64	R	>64	R
Piperacillin/Tazobactam	>16	R	>16	R
Tigecycline	2	S	2	S
Tobramycin	>4	R	>4	R
Trimethoprim/ Sulfamethoxazole	>4/76	R	>4/76	R
Aztreonam/Avibactam	0.19	S	0.25	S
Ceftazidime/Avibactam	0.75	S	32	R
Imipenem/Relebactam	0.75	S	16	R
Meropenem/Vaborbactam	0.50	S	32	R

**Table 3:** In-silico predictions for plasmid typing, including identification of replicon, relaxase and mate pair formation protein types. MOB types also predict mobility of a plasmid, such as conjugative, mobilizable and non-mobilizable. Features in **bold** denote common detection in both isolates. (Abbreviations. MOB types (MOBC, MOBF, MOBP): mobility types of relaxase proteins. MPF: mate pair formation protein types).

	Plasmid Type	Relaxase Type	MPF Type	Predicted Mobility
INT18S	ColRNAI, ColRNAI, ColRNAI, **IncFIB(K)**, IncFIB(pQil), IncX3	MOBC, **MOBF**, MOBP	**MPF_T**	Conjugative
INT20U	IncFIA(HI1), **IncFIB(K)**, IncFII(K), IncR	**MOBF**	**MPF_F**	Conjugative

**Table 4:** Genomic differences in INT18S and INT20U *K. pneumoniae* isolates based on yersiniabactin sequence types, lineage and allele numbers in ybt genetic loci. Predicted data were calculated by Kleborate v3.1.3.

	INT18S	INT20U
YbST	299-1LV	230-2LV
Yersiniabactin Lineage	Ybt13; ICEKp2	Ybt15; ICEKp11
Alleles		
*ybtS*	6	6
*ybtX*	4	15
*ybtQ*	20	22
*ybtP*	14	4
*ybtA*	1	1
*irp2*	220	37
*irp1*	131	34
*ybtU*	2	14
*ybtT*	4	1
*ybtE*	52	4
*fyuA*	2	17

**Table 5:** RT-PCR primers used to detect common carbapenem-resistant genes in the isolated strains.

TargetGene	Type	Name	Sequence (5′ → 3′)	5′ Label	3′ Label
*KPC*	Primer (forward)	KPC_fwd	GCAGCGGCAGCAGTTTGTTGATT	–	–
	Primer (reverse)	KPC_rev	GTAGACGGCCAACACAATAGGTGC	–	–
	Probe	KPC_probe	CAGTCGGAGACAAAACCGGAACCTGC	ROX	BHQ-2
NDM	Primer (forward)	NDM_fwd	CCAGCAAATGGAAACTGGCGAC	–	–
	Primer (reverse)	NDM_rev	ATCCAGTTGAGGATCTGGGCG	–	–
	Probe (ABI7500)	NDM_probe_ABI7500	ACCGAATGTCTGGCAGCACACTTC	TAM	BHQ-2
OXA-48	Primer (forward)	OXA48_fwd	GATTATGGTAATGAGGACATTTCGGGC	–	–
	Primer (reverse)	OXA48_rev	CATATCCATATTCATCGCAAAAAACCACAC	–	–
	Probe (ABI7500)	OXA48_probe_ABI7500	CCATTGGCTTCGGTCAGCATGGCTTGTTT	JOE	BHQ-1
VIM	Primer (forward)	VIM_fwd	TTGCTTTTGATTGATACAGCGTGGGG	–	–
	Primer (reverse)	VIM_rev	GTACGTTGCCACCCCAGCC	–	–
	Probe	VIM_probe	TCTCGCGGAGATTGAAAAGCAAATTGGACTTCC	CY5	BHQ-3

## Data Availability

The whole genomes of *K. pneumoniae* INT18S and INT20U were deposited in DDBJ/ENA/Genbank under the accession numbers JBRBEL000000000 for INT18S (https://www.ncbi.nlm.nih.gov/nuccore/JBRBEL000000000, BioProject ID: PRJNA1291179 accessed on 28 August 2025) and JBPXMH000000000, (https://www.ncbi.nlm.nih.gov/nuccore/JBPXMH000000000, BioProject ID: PRJNA1291402 accessed on 28 August 2025) for INT20U, respectively.
